# Spatial Assessment of Livestock Heat Stress in Thessaly Region of Greece Using ERA5-Land Reanalysis and Temperature–Humidity Index

**DOI:** 10.3390/vetsci13050434

**Published:** 2026-04-29

**Authors:** Vasileios G. Papatsiros, Eleftherios Chourdakis, Georgios Tsegas, Lampros Fotos, Georgios I. Papakonstantinou, Alexandra V. Michailidou, Dimitrios Gougoulis, Konstantina Dimoveli, Evangelos-Georgios Stampinas, Eleftherios Meletis, Irene Valasi, Christos Vlachokostas

**Affiliations:** 1 Clinic of Medicine, Faculty of Veterinary Medicine, School of Health Sciences, University of Thessaly, 43100 Karditsa, Greece; l.fotos@uth.gr (L.F.); geopapak@uth.gr (G.I.P.); dgoug@uth.gr (D.G.); kdimoveli@uth.gr (K.D.); elmeletis@uth.gr (E.M.); 2 Sustainability Engineering Laboratory, Energy Department, School of Mechanical Engineering, Aristotle University of Thessaloniki, 54124 Thessaloniki, Greece; chourdakis@auth.gr (E.C.); gtsegas@auth.gr (G.T.); amicha@meng.auth.gr (A.V.M.); vlahoco@meng.auth.gr (C.V.); 3 Laboratory of Physiology, Faculty of Veterinary Science, School of Health Sciences, University of Thessaly, Trikalon 224, 43100 Karditsa, Greece; estampinas@uth.gr

**Keywords:** climate change, ERA5-Land reanalysis, THI, Precision Livestock Farming, Digital Twin, farm

## Abstract

In the Mediterranean principality of Thessaly, Greece, heat stress has become an environmental limitation on animal production and welfare. This study aims to quantify livestock heat stress using the temperature–humidity index (THI) and assess its spatial and temporal distribution across Thessaly during the warm seasons from 2020 to 2025, based on ERA5-Land reanalysis data. For selected livestock units, hourly air temperatures and dew point temperatures were used to generate and calculate maximum temperature fields and the THI under outdoor conditions, with no directly measured physiological responses in animals, but potential heat stress exposure was evaluated using THI derived from ERA5-Land data. The results reveal persistent thermal hotspots in the central and southeastern Thessalian plain, where maximum daily temperatures frequently exceeded 38–40 °C and locally surpassed 45 °C during August. THI values regularly exceeded 72, indicating productivity decline, and reached 82 during peak summer months, corresponding to high and severe stress categories. Mountainous regions were consistently 6–10 °C cooler and exhibited lower THI levels. Thermally stressful conditions extended from May through September, indicating sustained seasonal exposure rather than isolated heatwave events. The spatial coincidence between intensive livestock production and high-THI zones suggests structural vulnerability under current climate conditions. These findings offer a spatially explicit assessment of climate-driven thermal risk and support the development of targeted mitigation strategies and climate-resilient livestock management in Mediterranean agricultural regions. They also offer a data-driven foundation for integration into emerging Digital Twin frameworks for predictive livestock management.

## Simple Summary

This study examines temperature and humidity extremes in the Thessaly Region of Greece from 2020 to 2025. The investigation aimed to identify areas and periods where livestock are mostly affected by heat stress. The results indicate that the relatively flat agricultural lowlands in the central and southeastern parts of Thessaly generally experience very hot summers, with maximum ambient temperatures during the summer months often reaching or exceeding 38 to 40 °C and occasionally rising above 45 °C. This intense heat likely has considerable negative effects on livestock performance, including health, welfare, and production. The mountainous areas of Thessaly are cooler than the lowlands and are, therefore, less susceptible to such conditions. The report also shows that the potential for livestock to experience heat stress increases significantly during high-density heatwave events from May to September each year, making livestock more vulnerable to prolonged heat stress. Therefore, livestock in the lowlands require specific adaptation strategies to protect their welfare and maintain sustainable production in response to climate change, including the development of more effective ventilation and cooling systems.

## 1. Introduction

Heat stress is widely recognized as one of the most critical environmental challenges affecting livestock production systems, particularly in regions characterized by hot and dry summers such as the Mediterranean basin. Elevated ambient temperatures, especially when combined with high humidity, disrupt thermoregulation, reduce feed intake and milk yield, impair reproductive performance and alter metabolic balance [1,2,3].

Recent climate assessments indicate increasing frequency and duration of heatwaves across southern Europe, leading to an extension of the effective summer season and increased thermal pressure on agricultural systems [4,5]. The Mediterranean region is considered a climate change hotspot, with rising summer temperatures and longer warm seasons posing significant challenges for livestock production [4,5,6]. These changes are especially critical for lowland agricultural areas, where reduced ventilation and high stocking densities can worsen heat accumulation and reduce animals’ ability to dissipate heat effectively [4,5,6].

Heat stress in livestock is a complex issue; animals must cope with severe biological challenges affecting their overall health due to systemic effects of heat stress [2]. For example, discrepancy in the temperature–humidity index (THI) has been shown to affect dry matter intake, rumen fermentation, and endocrine regulation, and cause nutritional partitioning to focus more on maintaining overall health rather than on production or reproduction [2]. Additionally, chronic exposure to heat stress increases an animal’s risk of developing mastitis, reproductive failures and metabolic disorders, especially in peripartum animals whose homeostasis is threatened by new metabolic demands [7,8].

Thessaly, one of Greece’s main agricultural regions, combines extensive lowland plains with a high density of livestock, making it particularly vulnerable to climate-driven heat stress [9]. Intensive dairy and ruminant systems are predominantly concentrated in the Thessalian plain, making spatial assessment of climate-driven thermal vulnerability particularly relevant [9].

Reanalysis datasets such as ERA5 have been widely applied in climate impact studies, including assessments of heat stress, agricultural risk, and livestock exposure to extreme temperatures [10,11,12]. These databases make it possible to assess the assessment of environmental conditions, heat stress, and the risks to agriculture and livestock from heat stress. However, although there is extensive research on the impacts of climate change on livestock, little attention has been given to evaluating heat stress at both regional and local levels using high-resolution reanalysis databases for the Mediterranean. In particular, the use of the ERA5-Land database together with livestock thermal stress indicators, such as the THI, has rarely been used to identify areas with consistent thermal stress and cumulative exposure.

Furthermore, the application of climate-based heat stress indicators in emerging concepts such as Precision Livestock Farming (PLF) and Digital Twin (DT) systems—which involve continuous monitoring of environmental and animal parameters for decision-making and prediction—requires further investigation.

To date, neither the spatial distribution of elevated summer temperatures in the Thessaly region of Greece nor their impact on the THI within livestock farms has been studied by means of ERA5-Land reanalysis data. Thus, the aim of this study was (i) to map the spatial pattern of maximum summer temperatures in Thessaly for 2020–2025 using the ERA5-Land reanalysis database; (ii) to calculate the THI to reflect potential livestock heat stress under outdoor conditions, with no direct measurement of physiological responses; and (iii) to identify areas with persistent thermal exposure for livestock systems.

## 2. Materials and Methods

### 2.1. Study Area

The Thessaly Region, located in central Greece, spans a total area of 14,036 km^2^ (approximately 11% of the national territory). The region’s topography is characterized by a dual landscape: extensive low-elevation agricultural plains (typically <200 m above sea level), centrally located, which are framed by a mountainous perimeter exceeding 1000 m in elevation. While the entire region exhibits a Mediterranean climate with hot, dry summers and mild winters, this study is mostly focused on the lowland Thessalian plain, where livestock production—particularly dairy cattle and small ruminants—is spatially concentrated.

### 2.2. ERA5-Land Reanalysis Data

The analysis is based on ERA5-Land reanalysis data [13,14] produced by the European Centre for Medium-Range Weather Forecasts (ECMWF). ERA5-Land provides hourly estimates of surface meteorological variables at a spatial resolution of 0.1° × 0.1°, ensuring temporal consistency and physical realism across long time periods. Hourly 2 m air temperature, measured at a height of 2 m above the surface, and dew point were obtained for May–September 2020–2025 at 0.1° spatial resolution.

The analysis represents aggregated multi-year averages (2020–2025), a common approach in climatological analyses to reduce interannual variability [15,16]. No bioinformatics software was used; ERA5-Land data processing was conducted using standard computational tools.

### 2.3. Temperature Analysis

Within the present analysis, maximum daily temperature fields were derived for each month and aggregated across the warm season, and spatial patterns were evaluated in relation to topography and livestock unit distribution. The core analysis focuses on the traditional summer months (June–August), during which thermal stress typically peaks. However, consistent with recent findings documenting the lengthening of the warm season in southern Europe [7,17], the months of May and September were also introduced in the calculation process. Relative humidity was derived from dew point using established psychrometric relationships [18].

The computational approach was further refined by integrating monthly and seasonal averages, and extreme-value parameters.

### 2.4. Temperature–Humidity Index (THI)

Thermal stress was measured using the THI, one of the most widely used methods for evaluating heat stress in animals [19,20]. The THI measures the combined effect of air temperature and relative humidity on the regulation of animal body temperature. The THI was calculated using the following formula:

THI = T_air_ − (0.55 − 0.055 × RH) × (T_air_ − 14.5) where THI is the temperature–humidity index, T_air_ is the air temperature (°C), and RH is the relative humidity (%). Dew point values were converted to relative humidity using standard psychrometric relationships [18].

THI was then computed as a function of air temperature and relative humidity. Solar radiation was excluded from formulation following recommendations for indoor or shaded environments where animals are not directly exposed to sunlight [21,22]. This formulation has been shown to provide reliable estimates of thermal discomfort in confined livestock systems and allows for robust comparisons among locations with different microclimatic characteristics [23].

THI was calculated at an hourly resolution using ERA5-Land data for each grid cell and livestock unit location. The resulting time series were then aggregated to derive the monthly, seasonal, and multi-year statistics. At this point, it should be noted that no in situ measurements were used in the frame of the present work. All the calculations are based on reanalysis data.

### 2.5. THI Classification

THI values were classified into five thermal stress categories based on thresholds commonly used in dairy and livestock research [24,25], enabling the identification of periods associated with reduced productivity and increased health risks (Table 1).

### 2.6. Farm-Scale Thermal Exposure Analysis Using ERA5-Land

To complement the regional-scale assessment of thermal exposure, a site-specific analysis was conducted focusing on the climatic conditions surrounding livestock units in Thessaly. For this purpose, ten livestock units participated in this study (Table 2). ERA5-Land reanalysis data for May–September 2020–2025 at a 0.1° spatial resolution were utilized to derive localized temperature fields for the period under consideration.

Hourly 2 m air temperatures and dew point temperatures were extracted and used to compute monthly and seasonal averages, and extreme temperature values. According to Section 2.4, thermal stress was measured using the THI.

This approach will establish the baseline thermal load on the environment of an animal kept indoors in a naturally ventilated building, allowing comparisons of thermal loads from various types of livestock facilities.

## 3. Results

### 3.1. Spatial Distribution of Maximum Summer Temperature (ERA5-Land, 2020–2025)

**Figure 1:**
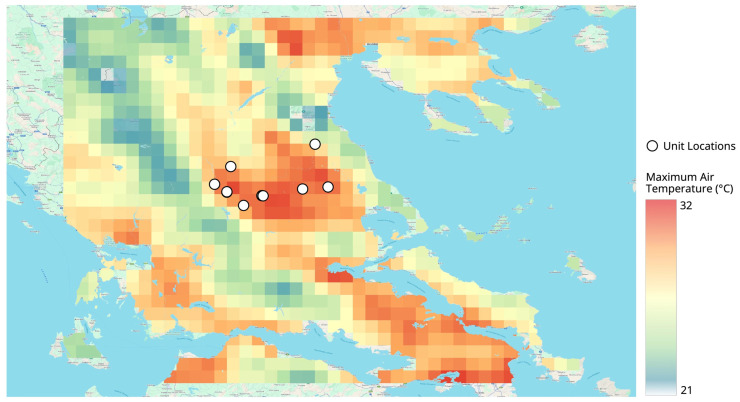
Spatial distribution of maximum daily air temperature (°C) over Thessaly based on ERA5-Land reanalysis data for the period 2020–2025. Maximum summer temperature aggregated over May–September.

**Figure 2:**
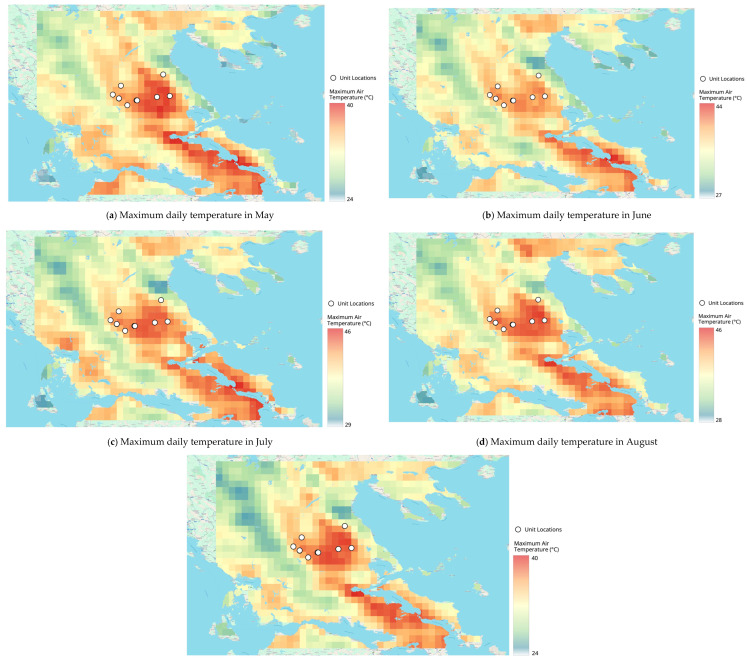
Monthly spatial distribution of maximum daily air temperature (°C) over Thessaly from ERA5-Land (2020–2025) during May–September. Panels (**a**–**e**) correspond to May–September, respectively. Livestock unit locations are overlaid.

Figure 1 presents a seasonal overview of maximum air temperature over Thessaly during the warm season (May–September) from 2020 to 2025 using the ERA5-Land reanalysis data. Figure 2a–e show monthly (May–September) distributions of maximum air temperature for the entire region. To assist with site-specific interpretation of the data, livestock unit locations were plotted on all the panels.

Persistent thermal hotspots in the central and southeastern lowlands of Thessaly indicate frequent temperatures exceeding 38 or 40 °C and occasionally reaching up to 45 °C in August, as measured with a thermometer. These persistent hotspots are attributed to the low elevation of the Thessalian plain, extensive agricultural land use and limited natural ventilation due to the low topographic elevation. In contrast, the northwestern and western mountainous regions, with the higher elevation, experience maximum temperatures generally 6 to 10 °C lower than those of the central and southeastern lowlands, highlighting the influence of both elevation and topographic characteristics on regional thermal conditions.

### 3.2. Seasonal Progression—Monthly Evolution of Maximum Temperature Patterns

Thermally stressful conditions were evident from May onward, when lowland maxima exceeded 36–38 °C. June and August showed expansion and intensification of high-temperature zones. September retained maxima above 40 °C in several lowland areas, indicating a delayed seasonal cooling. The seasonal pattern identified in Figure 1 is further resolved through the monthly panels (Figure 2a–e), which illustrate the temporal progression and persistence of thermal stress throughout the warm season. Already in May, maximum daily temperatures exceed 36–38 °C in parts of the central Thessalian plain, indicating an early onset of thermally stressful conditions.

Although elevated and mountainous areas remain comparatively cooler, several livestock units located in lowland zones are exposed to high temperature levels at the beginning of the warm season (Figure 2a). In June, both the intensity and spatial extent of high temperatures increase markedly. Maximum daily temperatures in the lowlands frequently exceed 39–40 °C, while cooler conditions become increasingly confined to mountainous areas. The contrast between lowland and elevated zones becomes more pronounced, indicating growing spatial heterogeneity in thermal exposure (Figure 2b).

July and August represent the peak of thermal stress across Thessaly. Maximum daily temperatures locally exceed 45 °C in the southeastern and central lowlands. Nearly all livestock units located within the Thessalian plain are embedded within zones of extreme heat, suggesting prolonged exposure to severe thermal conditions. The extensive area experiencing high temperatures indicates that there is limited synoptic or nighttime relief during this period (Figure 2c,d).

Although September has generally been cooler, it still presents a high thermal burden. The maximum lowland temperature exceeds 40 °C, demonstrating that the end of summer-like conditions will be delayed. There are very large temperature differences across extensive areas (Figure 2e).

### 3.3. Farm-Level Thermal Differentiation

**Figure 3:**
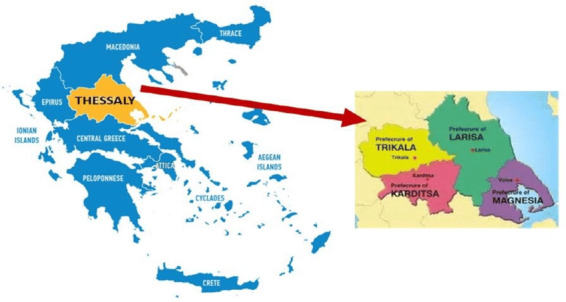
A map of Greece highlighting the region of Thessaly, which includes the four prefectures of Larisa, Magnesia, Trikala and Karditsa.

The localized ERA5-based analysis revealed pronounced spatial variability in thermal exposure across the livestock units, reflecting the strong influence of topography and proximity to water bodies on microclimatic conditions. Higher mean summer temperatures were consistently observed in lowland areas, particularly in the central and southeastern Thessalian plain, including the Larissa and Farsala regions (Larisa prefecture), and the Almyros–Nea Anchialos zone (Magnesia prefecture). In contrast, northwestern locations such as Elassona (Larisa prefecture), Kalambaka (Trikala prefecture) and Pyli (Trikala prefecture) exhibited considerably lower temperature levels (Figure 3).

**Figure 4:**
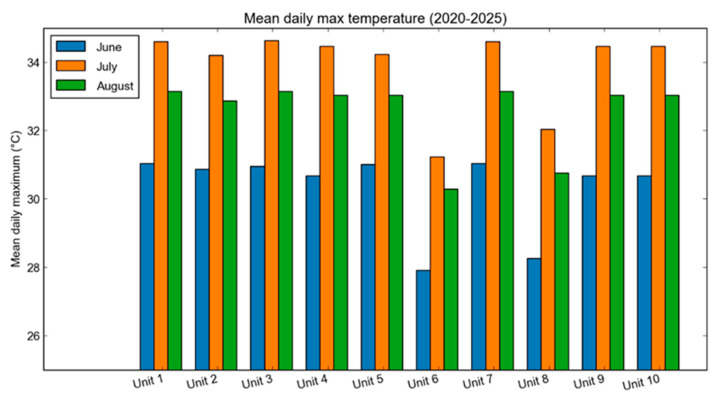
Mean maximum summer temperature at livestock unit locations for the core summer period (June–August) based on ERA5-Land data (2020–2025).

Differences between the warmest and coolest locations frequently exceeded 6–8 °C, confirming that livestock units located within the Thessalian plain are exposed to systematically higher thermal loads compared to those situated in elevated terrain (Figure 4). Figure 4 focuses on the core summer months (June–August), during which thermal stress peaks, while May and September are included in the broader seasonal assessment presented in Section 3.2 and Section 3.5.

Maximum daily summer temperatures at several farm locations regularly surpassed 35 °C and, in some cases, even approached or exceeded 40 °C. Diurnal temperature profiles indicate that peak thermal conditions typically occur between 14:00 and 17:00 local time, while nighttime temperatures often remain above 25 °C in lowland areas, potentially limiting nocturnal recovery from daytime heat load. Although proximity to water bodies may slightly moderate daytime maximum temperatures due to water’s thermal capacity, the associated increase in atmospheric humidity can elevate THI levels, thereby intensifying thermal discomfort despite the marginally lower air temperatures.

Due to the definition of THI, its monthly averages of daily maxima tend to accumulate to a narrow range of values showing little variation across units (Table 3).

### 3.4. Diurnal Thermal Profiles

**Figure 5:**
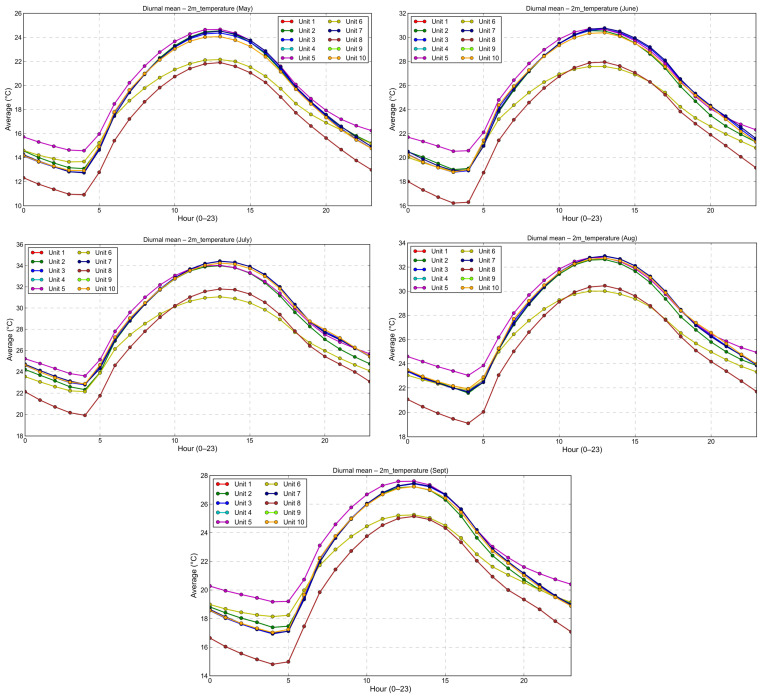
Mean daily temperature profiles from livestock units from May to September in the years 2020 to 2025.

Daily temperature cycles across the May–September period exhibit a consistent pattern across livestock locations (Figure 5). Morning temperatures remain relatively moderate (20–24 °C), followed by a rapid increase after 08:00 local time, when the sun reaches a certain angle. Peak values typically occur between midday and early afternoon (12:00–15:00), after which temperatures gradually decline. However, in several lowland locations, nocturnal temperatures remain elevated, frequently exceeding 25 °C (Figure 5). Such sustained high nighttime temperatures may impair the animal’s ability to shed heat that accumulates in its body; consequently, animals will experience increasing cumulative thermal stress rather than simply experiencing isolated daily thermal stress exposure.

### 3.5. THI Patterns

For much of the warm season, lowland agricultural areas recorded a THI greater than 72. During the peak summer months, significant portions of the Thessalian plain experienced temperatures exceeding 79–82 degrees. Areas at higher elevations had much less exposure to elevated THI. Livestock units in lowland areas were continuously exposed to moderate to severe THI levels.

Table 3 provides more detailed calculations of THI exposure at the livestock unit level, including total hours of exposure and the percentage of time spent within each THI stress level category (May–September 2020–2025). The results from Table 4 indicate that, while a substantial percentage of time was spent below the threshold for thermal stress, a significant proportion occurred within moderate to severe thermal stress, particularly at lowland livestock units. There were many instances where livestock units experienced “severe thermal stress” (THI > 82) for approximately 20% of the total exposure time, demonstrating the persistent and cumulative nature of the thermal load to which livestock were subjected during the warm season.

### 3.6. Implications for Livestock Unit Exposure

Figure 1 and Figure 2a–e illustrate both the geographical relationship between the location of livestock units (in higher temperature areas) and the thermal characteristics of these regions as identified by the data. In the Thessalian plain, livestock units are consistently exposed to maximum temperatures from early summer to early autumn, which are comparatively higher than those experienced by livestock units in elevated or more complex topographical areas. Some exceptions occur where the proximity of livestock units to coastal or inland bodies of water moderates maximum air temperatures; however, these potential benefits may be offset by higher atmospheric relative humidity, which could increase thermal discomfort when assessed using combined temperature–humidity indices. Therefore, the maximum temperature fields developed from the data presented in Figure 1 and Figure 2 form the basis for understanding how subsequent thermal stress analyses, based on the THI, can be interpreted.

## 4. Discussion

This study represents the first investigation into how the spatial distribution of maximum summer temperatures, using ERA5-reanalysis data, affects the livestock systems in the Thessaly Region over the past several years (2020–2025). A clear and ongoing trend of increased risk of heat stress is indicated throughout the Thessaly Region. However, there is substantial variability between different parts of the region (i.e., it is highly heterogeneous within the region), and extended periods during which livestock are exposed to thermal stress (i.e., many months). Based on the results of the current research, it can be concluded that heat stress in livestock operations in the Thessaly region is not an episodic phenomenon but a continuous and spatially organized environmental factor affecting these operations. The analysis of maximum temperature fields and THI values shows that livestock operations in low-lying areas experience prolonged periods of thermal stress, while mountainous locations are characterized by lower levels of thermal load. In this context, the present study achieves its objective by quantitatively analyzing the thermal stress levels in livestock operations and highlighting the crucial role of geography and seasonality in the exposure of livestock systems to heat stress. Notably, this research contributes to the identification of areas of persistent thermal exposure for Thessalian livestock systems, paving the way for integration into Precision Livestock Farming (PLF) and Digital Twin (DT) applications.

The central and southeastern lowlands consistently exhibit the highest thermal load across all five months of this analysis (May to September). Maximum daily mean temperatures range from 37 to 40 °C, with the highest values during the peak summer months (August) reaching 45 to 46 °C in parts of the Thessalian plain. These temperatures are significantly higher than the established thermoregulation thresholds for livestock, regardless of whether animals are housed indoors or under shade, and can negatively affect feed intake and reproductive performance and increase the incidence of metabolic or other health-related disorders in livestock [1,2,9]. In comparison, maximum temperatures in the northwestern and western mountainous areas are typically 6 to 10 °C lower than those experienced during peak summer periods. The temperature differences between the northern and southern extreme locations demonstrate that elevation is a primary factor influencing the risk of heat stress. The cooling effect of elevation reduces both the sensible heat load and the retention of nighttime temperatures, allowing livestock to recover from a thermoregulation perspective during the night. Similar spatial patterns have been documented for Mediterranean livestock systems, with mountainous regions providing partial summer refuge from extreme heat [3,26].

Humidity affects the performance of heat dissipation by evaporation, particularly when confined housing systems limit air movement [22]. As solar radiation was excluded from the current THI calculation to simulate production environments found indoors, the results from the current THI are directly applicable to intensive dairy and ruminant systems typical of the area. These findings emphasize the need to consider temperature and humidity metrics when developing spatial risk assessment tools, especially in lowland regions where intensive agriculture exists [4,5]. When humidity was included in calculating THI, values often exceeded the threshold at which production declines are expected (THI > 72). Particularly, during peak summer months, values could reach >79–82, indicating a high degree and severe category of stress. These findings are supported by evidence of increasing air temperature with increasing humidity and a progressively lengthening warm season, in accordance with climate trends within the Mediterranean basin [4,5]. Biologically, the conditions described above exceed the upper critical temperatures that can be tolerated by high-producing dairy cattle and intensively managed ruminants. Reduced dry matter intake due to exposure to THI > 72 has been linked to lower milk production and changes in rumen function [1,24]. Exposure to THI > 79 causes an increased physiological load on animals, causing increased respiratory rates, vasodilation of peripheral blood vessels and redistribution of blood from visceral organs to the skin surface. Sustained activity of these thermoregulatory mechanisms that maintain temperature increases the energy required for maintenance and decreases the amount of nutrients allocated to productive activities. Thus the overall productive efficiency of animals decreases.

The key result from this research shows that elevated maximum temperatures and high THI values were sustained over a long period from early summer to September as a cumulative, rather than episodic, event. Previous experimental and field research suggests that inadequate nocturnal cooling impairs recovery of core body temperature and exacerbates negative energy balance [20]. Consequently, the prolonged duration of elevated THI identified here increases the likelihood of chronic metabolic stress, particularly in high-yielding animals. Furthermore, the marked spatial contrast between warmed lowland zones and comparatively cooler elevated areas underscores the need for targeted and location-specific adaptation strategies in livestock management.

Under continued regional warming, exceedance of moderate and severe THI thresholds is likely to occur earlier in spring and persist later into autumn, further increasing cumulative annual heat load. Heat stress in Thessaly can therefore be characterized as persistent across an extended warm season, spatially concentrated in lowland agricultural zones. Livestock systems in the Thessalian plain are already operating close to or beyond thermal comfort limits for prolonged periods each year. In addition to affecting productivity, sustained exposure to high THI levels has welfare implications, including altered lying behavior and reduced rumination. Moreover, prolonged exposure to high THI levels has been shown to increase cortisol levels and reduce immune competence [2,9]. At extremely high THI levels of THI (>82), particularly during multi-day extreme heat events, mortality has been shown to increase [25]. The metabolic and endocrine adjustments required to maintain homeothermy under such conditions—including altered insulin, cortisol, and thyroid hormone signaling—can further impair reproductive efficiency [27]. Therefore, thermal stress represents a systemic biological challenge affecting metabolism, immunity, reproduction, and overall animal welfare. Adaptation measures such as enhanced ventilation, evaporative cooling systems, improved housing design, and genetic selection for thermotolerance will become increasingly significant. Economic analyses have shown that heat stress imposes substantial financial losses on livestock industries [28], and similar pressures are likely to intensify in Mediterranean production systems.

In this context, the findings of the present study are closely aligned with broader sustainable development objectives. The spatially explicit identification of thermally vulnerable sub-regions supports the Sustainable Development Goal 13 (SDG 13-Climate Action) of the United Nations by providing regional-scale evidence of climate change impacts and informing targeted adaptation strategies [29,30]. The implications for livestock productivity and food system stability directly relate to SDG 2 (Zero Hunger), as climate-induced heat stress has been shown to reduce animal performance, reproduction, feed efficiency and productivity of farm animals [26,31]. At the same time, climate-informed and site-specific mitigation measures contribute to SDG 12 (Responsible Consumption and Production) by promoting improved resource efficiency and sustainable livestock management [32,33]. Improvements in thermal comfort conditions are indirectly linked to SDG 3 (Good Health and Well-being), as reduced heat stress enhances animal welfare and resilience [34]. Finally, the integration of reanalysis-based climate intelligence into livestock management frameworks also aligns with SDG 9 (Industry, Innovation and Infrastructure), by encouraging innovation, digital climate services, and climate-resilient agricultural systems [30,35].

**Figure 6:**
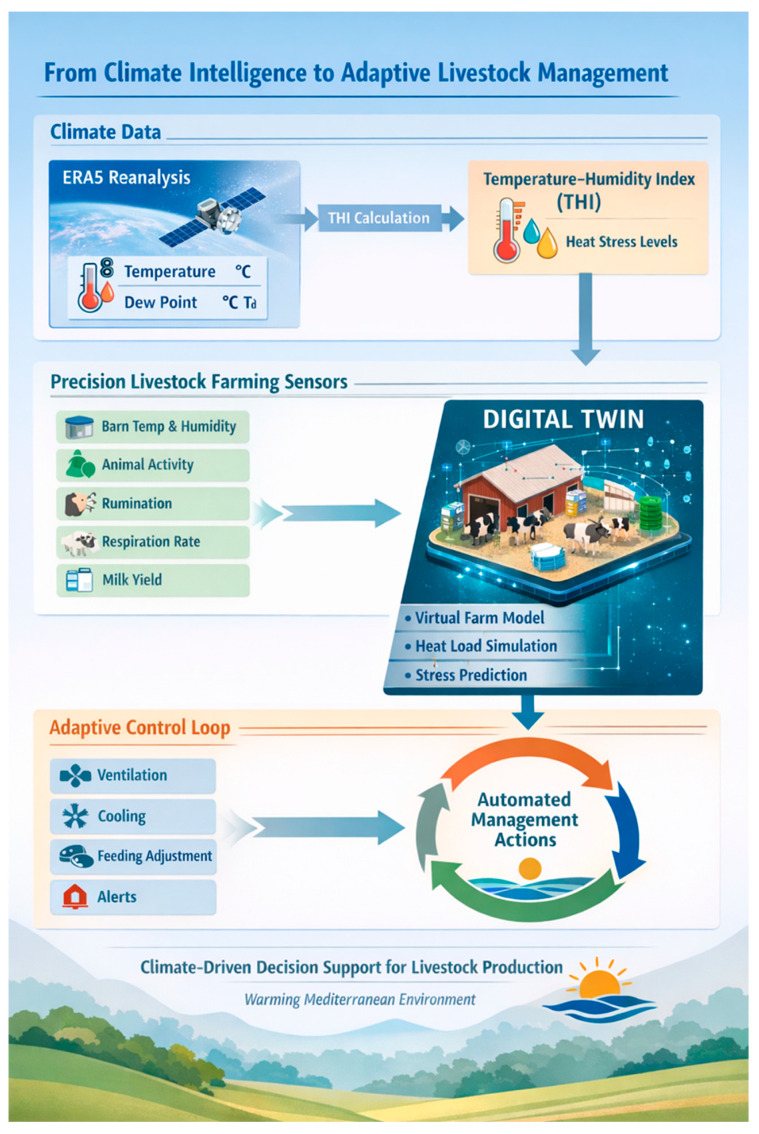
The Precision Livestock Farming (PLF) and Digital Twin (DT) frameworks use a conceptual architecture to integrate climate intelligence derived from reanalysis (ERA5-Land data). These datasets can generate several thermal indicators, including the temperature–humidity index (THI). Input from these environmental datasets interacts with monitoring data from various levels (animal, barn, etc.) within three distinct DT components: Environmental Twin, Animal Twin and Barn Twin.

The spatially explicit quantification of thermal exposure across the livestock units of our study provides a robust foundation for integration into PLF systems and emerging DT architectures. PLF is based on continuous, automated monitoring of animal—and environment-level parameters to improve management efficiency, welfare, and sustainability [36,37,38]. By coupling ERA5-derived climatic indicators [13,14] and THI-based stress thresholds [19,20] with real-time on-farm sensor data, it becomes possible to construct dynamic, site-specific thermal risk models. In parallel with advances in climate data analytics, emerging digital approaches such as DT systems are increasingly being explored in livestock production [39]. Within a DT framework, each livestock unit can be represented as a continuously updated virtual replica that assimilates environmental drivers and physiological responses to simulate heat-stress trajectories and predict performance deviations [39,40]. Due to the nonlinear nature of heat stress, which increases over time with persistent exposure [2,22], it is important to incorporate cumulative climate indicators into DT models so that subclinical heat stress can be detected before productivity loss occurs. Consequently, the climate analyses in this study go beyond examining the historical profile of a region’s vulnerability to climate stresses; they provide a scalable, data-driven (bottom-up approach) framework for climate-informed decision making. As the region warms due to climate change [4,5], integrating reanalysis-based climate intelligence into PLF and DT systems will become necessary to sustain production, protect animal welfare and ensure the long-term resilience of systems (Figure 6). To develop a DT framework for livestock systems in Thessaly, a spatial and seasonal assessment of the region’s thermal conditions is required based on a DT architecture, consisting of the following inter-related systems:

(a) The Environmental Twin: It incorporates hourly ERA5 climate data and THI outputs to generate 24–72 h forecasts, quantify exceedance hours above critical thermal thresholds and assess cumulative heat load, reflecting patterns of elevated THI and persistent heatwaves.
(b) The Animal Twin: It represents the physiological state of the animals by combining measurements such as respiration rate, behavioral activity, feed intake and milk yield with predicted thermal burden [2,41].
(c) The Barn Twin: It simulates housing level conditions, modeling airflow, ventilation performance, cooling-system efficiency and humidity, in agreement with management strategies known to reduce body heat load such as forced ventilation, shading and evaporative cooling [1].

Overall, assessing spatial and seasonal thermal vulnerability in livestock systems represents a multidisciplinary contribution bridging climate science, animal physiology, and sustainable agricultural development. Despite the small number of livestock units that participated in the study, analysis of individual farms demonstrates marked differences in the thermal environment of each farm due to local and site-specific geographic influences (i.e., elevation, ventilation potential and proximity to bodies of water). Future studies that correlate climatic indicators with farm productivity data such as milk yields, fertility rates and morbidity statistics will help define the management and economic outcomes of the identified thermal patterns. The increase in the number of units experiencing “mild” to “hot” THI conditions in the latter part of the study indicates a rise in summer thermal loads at the farm level and highlights the need for targeted adaptive management techniques, such as improved ventilation, shading or cooling systems.

Finaly, this study emphasized that, especially for Greece, adaptation strategies related to sustainable livestock systems should be meticulously considered, in a holistic framework that includes other adaptation options for the agricultural/farming sector and sustainable animal and plant production, i.e., rational water management, integrated cultural management methods, promoting bioenergy alternatives from livestock waste, improved forest management, and other strategies such as promotion of sustainable agri-food tourism strategies [42].

The following limitations apply to the above-mentioned study. Firstly, although the ERA5-Land database provides high-frequency data, its low spatial resolution (0.1°) does not allow detailed consideration of microclimatic changes at the farm scale. Secondly, the THI calculation formula used ignores solar radiation; therefore, heat stress under sunlight may be underestimated. Thirdly, as only reanalysis data are considered in this study, no farm-specific management information is included. In future research, in situ observations and physiological animal data should be considered.

## 5. Conclusions

This study provides a spatially explicit assessment of climate-driven thermal vulnerability in livestock systems across the Thessaly Region of Greece, during the 2020–2025 warm seasons, integrating ERA5-Land reanalysis data with a THI framework. Heat stress in the region is not confined to episodic extreme events but constitutes a persistent and seasonally prolonged environmental constraint, especially in lowland areas of the Thessalian plain. Notably, farm-level ERA5-Land analysis shows substantial variation in heat exposure due to factors like elevation, water proximity, and ventilation, resulting in diverse heat stress levels. While some areas remain below critical thresholds, others face prolonged moderate-to-severe heat load, harming the productivity and welfare of farm animals. Hence, integrating localized climatic intelligence into on-farm management decisions can support more effective adaptation and mitigation measures and enhance the resilience of livestock production in Mediterranean livestock systems under ongoing warming trends.

Future research should include high-resolution in situ measurements of both meteorological and animal physiological variables at livestock farms, as this will improve the precision of heat stress risk assessment beyond the current reanalysis approach. The use of in situ sensors to continuously monitor animal-level responses to heat stress within a PLF framework would be ideal. Additionally, research aimed at determining the impact of solar radiation, wind speed, and shelter design can significantly enhance the development of improved heat stress index models. Studies linking THI levels with production, health, and reproductive performance are also necessary to understand the implications for economic gains and losses.

## Figures and Tables

**Table 1:** THI classification.

THI Range	Stress Level	Description
<68	Below stress	No or negligible thermal stress
68–72	Mild stress	Slight thermal discomfort
73–78	Moderate stress	Possible reduction in productivity
79–82	High stress	Severe impact on comfort and performance
>82	Severe stress	Extreme stress, high health risk

**Table 2:** Livestock unit locations.

Livestock Unit	Animal Species	Location (HGRS87)		Prefecture of Thessaly
		Χ (m)	Υ (m)	
1	Sheep and goats	330,940	4,366,719	Trikala
2	Cattle and fattening calves	367,257	4,411,628	Larisa
3	Sheep and goats	317,415	4,358,561	Karditsa
4	Sheep and goats	330,111	4,366,887	Karditsa
5	Cattle–dairy cows	298,024	4,377,705	Trikala
6	Cattle–dairy cows	306,218	4,370,832	Trikala
7	Pigs	358,057	4,372,217	Larisa
8	Cattle–dairy cows	375,518	4,373,718	Larisa
9	Pigs	306,249	4,370,517	Larisa
10	Sheep and goats	309,481	4,393,020	Karditsa

**Table 3:** Mean daily max THI for the core summer period (June–August) based on ERA5-Land data (2020–2025).

Unit	Months		
	June	July	August
1	96	103	94
2	89	96	89
3	95	103	94
4	94	103	94
5	96	103	94
6	96	103	94
7	94	102	92
8	88	95	87
9	96	104	95
10	94	103	94

**Table 4:** The distribution of overall thermal stress experienced by the ten livestock units by THI category from May to September in the years 2020 to 2025. The total number of hours and relative percentages for each livestock unit in each THI category were summed to obtain the THI category distribution. The thermal stress categories reflect the thresholds set for each category in Table 1.

Unit	Total Number of Hours and Percentages	Thermal Stress Level				
		Below	Mild	Moderate	High	Severe
1	Number of hours	5323	537	761	469	1670
	Percentages	61%	6%	9%	5%	19%
2	Number of hours	5529	482	850	559	1340
	Percentages	63%	6%	10%	6%	15%
3	Number of hours	5303	553	800	462	1642
	Percentages	61%	6%	9%	5%	19%
4	Number of hours	5309	554	816	479	1602
	Percentages	61%	6%	9%	5%	18%
5	Number of hours	5323	537	761	469	1670
	Percentages	61%	6%	9%	5%	19%
6	Number of hours	5323	537	761	469	1670
	Percentages	61%	6%	9%	5%	19%
7	Number of hours	5342	564	851	485	1518
	Percentages	61%	6%	10%	6%	17%
8	Number of hours	5962	616	744	403	1035
	Percentages	68%	7%	8%	5%	12%
9	Number of hours	5122	499	757	552	1830
	Percentages	58%	6%	9%	6%	21%
10	Number of hours	5309	554	816	479	1602
	Percentages	61%	6%	9%	5%	18%

## Data Availability

The original contributions presented in this study are included in the article. Further inquiries can be directed to the corresponding authors.

## Abbreviations

The following abbreviations are used in this manuscript:

DT	Digital Twin
ECMWF	European Centre for Medium-Range Weather Forecasts
PLF	Precision Livestock Farming
RH	Relative Humidity
SDG	Sustainable Development Goal
T_air_	Air temperature in degrees °C
THI	Temperature–Humidity Index
